# Health-Related Challenges and Coping Strategies Among Women During Pandemics: A Systematic Review of Qualitative Studies

**DOI:** 10.3389/frhs.2022.847753

**Published:** 2022-04-15

**Authors:** Mili Roopchand Sahay, Shubhankar Dubey, Rakesh Kumar Sahoo, Srikanta Kanungo, Krushna Chandra Sahoo, Sanghamitra Pati

**Affiliations:** ICMR-Regional Medical Research Centre, Bhubaneswar, India

**Keywords:** gender, women, vulnerability, pandemic, SDGs

## Abstract

Equality and empowerment for women are among the 17 Sustainable Development Goals (SDGs 5). Although women were confronted with more challenges in various ways during pandemics; however, there is hardly any systematic synthesis of evidence on women's health-related challenges during pandemics. We reviewed the health challenges faced by women during the pandemic. We searched MEDLINE, PsycINFO, and CINAHL following PRISMA guidelines. We identified 2,831 studies, of which we included 17. Reproductive health, psychosocial health, and gender-based violence emerged as significant challenges. Many studies reported challenges in provisions for routine services and increased anxiety, fear, and stress among women. The findings highlighted that pandemic have a significant impact on women's health. Women must have equal rights and opportunities without discrimination, which requires urgent action to enhance women's rights and to achieve SDGs. Women engagement/involvement in pandemic-related services needs to be explored, which will aid in developing strategies to alleviate vulnerabilities.

## Introduction

Several pandemics have occurred throughout human history, most recently those caused by smallpox, cholera, plague, influenza, Ebola, severe acute respiratory syndrome (SARS), West Nile disease, and COVID-19 (1, 2). Pandemics have infected millions of people, resulting in widespread illness and thousands of deaths (3, 4). Apart from the debilitating and sometimes fatal effects on those directly affected, pandemics have a slew of negative social, economic, and political consequences (5, 6). Pandemics have enormous ramifications, particularly for women (7–9).

The pandemics have a systemic problem of human development that exacerbates challenges to gender equality (10, 11). The crisis disproportionately impacts women and girls on various social, economic, and political levels. Women lost jobs more rapidly than men, owing to the high percentage of women employed in hard-hit businesses such as restaurants and hotels. Women working in the informal economy have suffered from a lack of health care, unemployment benefits, and other protections (12). Pandemics are having an effect on gender disparity in health and education and the burden of unpaid care work and gender-based violence. While the pandemics affects everyone, women and girls suffer particular and frequently disproportionate economic, health, and social risks due to ingrained inequities, social norms, and unequal power relations (13). Understanding the gender-disaggregated effects of the pandemics crisis through sex-disaggregated data is crucial for developing policy solutions that minimize vulnerable conditions and improve women's agency while prioritizing gender equality (14). It employs impact evaluation research to identify policy alternatives to increase women's economic resilience and reduce any negative repercussions during the pandemics.

SDGs (Sustainable Development Goals) prioritize gender equality and empowerment and the elimination of all forms of discrimination against women (15). Despite progress toward gender equality, disparities persist in the form of sexual or intimate partner violence and social underrepresentation (16, 17). The difference becomes even more pronounced during unprecedented pandemics when women face adversity in the form of illnesses, economic burden, workplace or household stress, and more (18–20). Additionally, if a woman works, she must balance domestic responsibilities with professional responsibilities (21). Usually, women provided 70% of all health and social services staff globally (17). Moreover, the isolation and confinement may increase gender-based violence (22). Discord between women's personal and professional lives during a pandemic may have long-term consequences (23, 24).

Women generally have more experience using health facilities, whereas men may regard healthcare venues as “feminized” and frequently have low health literacy. This study provides an evidence-based overview of critical analysis of masculinities and pandemics in a gendered lens. It examines the impact of pandemics on men and women in various social groupings. It makes suggestions and recommendations to politicians and other decision-makers on addressing masculinity concerns during times of crisis. Pandemics exacerbate inequalities in women's health (12). Women faced additional obstacles in various ways during pandemics; however, there is less evidence focusing exclusively on women's health-related barriers. This study narrated the health challenges and coping women faced during pandemics.

## Methods

A systematic review of the existing literature was conducted, emphasizing health-related challenges and coping strategies among women during pandemics. We registered the protocol in PROSPERO (CRD42020187826), and the findings were reported following PRISMA guidelines (25). We conducted a thorough search of the MEDLINE (PubMed), PsycINFO (ProQuest), and CINAHL (EBSCO) databases to identify studies published between January 2000 and September 30, 2020.

We identified qualitative studies that documented the difficulties faced by women during pandemics. The titles and abstracts of the articles retrieved from the database were screened. The full text of the studies was independently screened by the authors (MRS and SD), and KCS resolved any disagreements. We included studies involving women of any age group. We excluded commentary, reviews and studies that did not meet the objective.

The authors (MRS, SD) independently extracted data from the included studies, with disagreements resolved by consulting a third reviewer (KCS). The Consolidated Criteria for Reporting Qualitative Research (COREQ) was used to assess the quality of studies (26) by two authors (MRS, SD) independently (Supplementary Material 1). We used thematic analysis (27) to synthesize the data and identify recurrent themes. The authors read and reread each article to identify potential codes, categories, and themes, as well as coded data, using MAXQDA Analytics Pro 2020 (VERBI GmbH Berlin).

## Results

**Figure 1:**
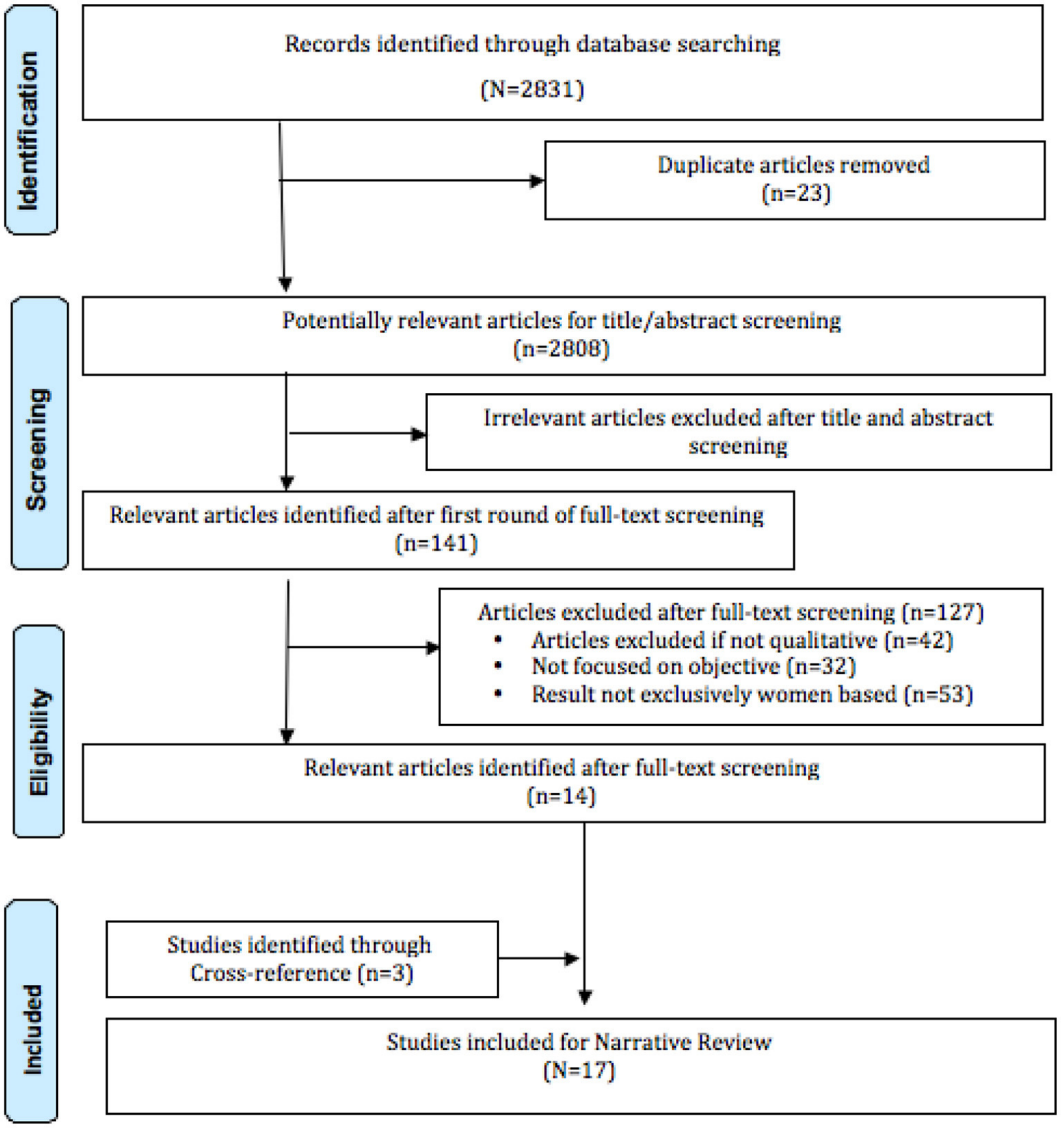
PRISMA flow diagram.

After screening 2,831 abstracts, 141 were considered relevant for full-text review, and finally, 17 were included (Figure 1). Included articles were on influenza (*n* = 3), COVID-19 (*n* = 5), Zika (*n* = 5), Ebola (*n* = 2) and SARS (*n* = 2). Eight studies reported reproductive health, while the remaining seven explored primarily psychosocial health (Table 1). The major themes were (1) Reproductive health, (2) Psychosocial health, and (3) Coping strategies and support systems (Table 2).

**Table 1:** Characteristics of the selected studies.

**Reference**	**Setting**	**Pandemic**	**Study participants**	**Data collection method**	**Analysis method**	**Major topic discussed**
Anderson et al. (28)	Brazil	Zika	Exclusively women	Semi-structured interviews	Content analysis	Reproductive health
Linde-Arias et al. (29)	Brazil, Puerto Rico, and the United States.	Zika	Exclusively women	Semi-structured interviews	Thematic analysis	Psychosocial and reproductive health
Belizan et al. (30)	Tegucigalpa, Honduras	Zika	More than half of the participants women	Semi-structured interviews and focus groups	Thematic analysis	Reproductive health
Boyd, (31)	Georgia	H1N1	Exclusively women	Focus group discussion	Thematic analysis	Psychosocial health
Chiang et al. (32)	Not mentioned	SARS	Exclusively women	Focus group discussion	Thematic analysis	Psychosocial health
Dodgson et al. (33)	Hong Kong	SARS	Exclusively women	Interview	Thematic analysis	Psychosocial and reproductive health
Erland and Dahl (34)	Sierra Leone	Ebola	Exclusively women	Semi-structured interviews	Thematic analysis	Reproductive health
Flowers et al. (35)	Glasgow	H1N1	More than half of the participants women	Focus group discussion, semi-structured interview	Thematic analysis	Psychosocial health
Gomez et al. (36)	Colombia	Zika virus	Exclusively women	In-Depth Interviews	Thematic analysis	Reproductive health
Jones et al. (37)	Sierra Leone	Ebola	Midwives, Medical staffs and Program Managers	In-Depth Interviews (*n* = 66)	Framework analysis	Reproductive health
Homer et al., (38)	Australia	COVID-19	Privately practicing midwives (all women)	Online bespoke survey	Content analysis	Reproductive health
Linde and Siqueira (39)	Boston	Zika	Exclusively women	Semi-structured Interviews	Content analysis	Psychosocial health
Lynch et al. (40)	Atlanta, Georgia, Dallas, Texas, Portland, Oregon	H1N1	Exclusively women	Focus group discussion	Framework analysis	Reproductive health
Sun et al., (41)	China	COVID-19	Two-third women participants	Face-to-face, Telephone interview	Framework analysis	Psychosocial health
Sabri et al. (42)	Africa, Asia, and Latin America	COVID-19	Exclusively women	In-depth interviews and key informant interview	Thematic analysis	Gender based violence
Tirado et al. (43)	Colombia	Zika	Exclusively women	Semi structured interviews	Content analysis	Reproductive health
Williams et al. (44)	United Kingdom	COVID-19	More than half of the participants women	Focus Group Discussion	Thematic analysis	Psychosocial health

**Table 2:** Summary of key findings.

**Pandemics**	**Reproductive health**	**Psychosocial health**	**Coping strategies and support systems**
COVID-19	• Change in consulting practices • Concerned about restriction • Difficulties in getting personnel protective equipment • Increased demand of homebirth • Lack of assistance during transport services • Lack of diagnostic services • Limited antenatal appointment • Limited consultation time	• Abusive relationships • Anxiety • Claustrophobia • Depression • Excessive media broadcasting • Family conflicts • Fear of infection • Fear of job loss • Stress due to loss of job • Financial hardship • Gender-based violence • Intimate partner violence • Loss of structure and routine	• Actively engaged in breath relaxation, music and regular exercise • Adjusted sleep • Engage with social media • Maintained physical strength • Professional responsibilities • Psychological defense mechanisms – speculation, isolation, distraction, self-consciousness, humor, rationalization • Reward and welfare system to motivate hospitals workers • Self-encourage • Social interaction
Severe acute respiratory syndrome (SARS)	• Disrupted counseling services • Family members not allowed to visit baby/mother after delivery • Inadequate counseling during pregnancy • Lack of routine care • Out of pocket expenditure	• Doubtful and confused • Feeling of separation due to changing norm • Living with uncertainty • Sleeplessness due to fear • Financial stress • Forced social isolation • Isolated from health care providers	• Change in daily routine • Halt on social activities • Self-mirroring • Self-preservation • Self-transcendence
Zika	• Doubtful about new pregnancy • Inadequate scientific information • Increased waiting time at facilities • Limited contraceptive services • Out of pocket expenditure • Poor access to specialist • Poor quality health services • Preferred for homebirth • Preferred private hospitals • Unavailability of diagnostic services	• Emotional wellbeing • Feeling discrimination • Feeling isolation • Less interaction with others • Mistrust • Sadness • Social stigma • Uncertainty • Uneasiness • Worried for adverse birth outcomes	• Avoided information about pandemics • Intense vigilance • Changed life style • Family support and happiness • Limited leisure and outdoor activities • Received support from family • Small support networks • Streaming from individual initiatives • Supported other for stress relief • Adherence prevention guideline
Ebola	• Demotivated toward institutional deliveries • Disrupted counseling services • Inadequate infrastructure for delivery • Lack of basic facilities in hospital • Lack of staff training on infection prevention • Misinformation about epidemic • Poor ambulance/referral services • Shortage of drugs • Unavailability of authority	• Fear of infection • Mistrust in midwives kept them away from health facilities • Rumors deterred people from visiting hospitals • Strained relationship between partners	• Active engagement of community • Competency in learning • Concern for family • Emphasized the need for a coordinated approach • Hiding their job with parents • Involvement of local health teams • Post EVD
H1N1 Influenza	• Inadequate scientific information	• Perception of risk was influenced by their personal proximity of care • Unfolded fear by media	• Adaptation of preventive behavior • Attitude of avoidance • Avoiding travel and travelers • Community sensitization

The studies were conducted in Africa, Asia, Australia, Brazil, China, Colombia, Georgia, Honduras, Hong Kong, Latin America, Puerto Rico, Scotland, Sierra Leone, the United Kingdom, and the United States. In 11 studies, the participants were exclusively women; in the remaining studies, more than half of the participants were women. The study participants were female community members and female healthcare professionals. All the studies were qualitative and collected semi-structured interviews or focus group discussions.

### Reproductive Health

Inadequate information and knowledge about pandemics, limited access to routine care, shift work for regular service providers, a shortage of medical facilities, and increased out-of-pocket expenses were all identified as common challenges during all types of pandemics (28, 33, 34, 37, 40, 41, 44). Many women and their family members believed hospitals were a source of infection during various pandemics and avoided hospital visits to avoid infection (34, 37). Women abstained from conception during pandemics out of fear or concern for inadequate prenatal care (28, 36, 39, 43). Additionally, many women were dissatisfied with institutional deliveries and preferred home delivery (28, 38). Some women opted for private facilities, increasing their out-of-pocket expenses (30, 33, 36, 37). Similarly, female healthcare providers stated that they felt helpless to treat patients due to insufficient infrastructure and supply, including essential equipment (41). Most of the time, medicines were out of stock, referral services were inadequate due to the absence of ambulances assigned to pandemic duties, and health care providers struggled to promote facility-based deliveries (28, 30, 37). During pandemics, new hospital policies limited interaction between intra-natal women and their families during their hospitalization (30, 33, 38). Additionally, women viewed public transportation as risky, preferring to travel in their vehicle or a hired taxi (33).

#### COVID-19 and SARS

Concerns about infection containment prompted restrictive practices such as limiting antenatal appointments, modifying consultation practices (telephonic consultation), and restricting consultation time in health facilities during COVID-19 (38). During the SARS (severe acute respiratory syndrome) epidemic, women's reproductive health was harmed by inadequate counseling services and isolation from healthcare providers (33).

#### Zika

Many pregnant women were concerned about abnormal deliveries, fearful that their children would be born with disabilities. They chose private facilities due to the lengthy wait for a doctor's appointment in public hospitals resulted in out-of-pocket expenses (28, 36). Additionally, due to the lack of family planning services, condom use was reported to be the only method of contraception available (28).

#### Ebola

The women faced difficulties such as disrupted counseling services, a lack of basic healthcare facilities, a drug shortage, and referral services (37). During the Ebola outbreak, there was a staff shortage due to relocation, trained infection control personnel, and personal protective equipment. Additionally, it was reported that authorities lacked adequate scientific information and accountability (34, 37).

#### H1N1 Influenza

The primary impediment to vaccine uptake was the lack of scientific information about vaccines (31). They believed that seasonal flu vaccination was a more secure option than the H1N1 vaccine (40). Women were encouraged to get vaccinated out of concern for their children's health (31, 35, 40).

### Psychosocial Health

The majority of studies have revealed that pandemics have a detrimental effect on women's psychological health. The most frequently reported psychological effects were fear, stress, anxiety, uneasiness, helplessness, and sadness. These effects had a detrimental impact on women's emotional wellbeing, exacerbating their concern for their families' safety, particularly the elderly and children (29, 31, 32, 34, 37–39, 41, 43, 44). Numerous studies have discovered that female health care providers face emotional stress daily while performing their duties (29, 32). Inadequate information about the new pandemic, a lack of effective pandemic management strategies, and work overload caused them to feel uneasy and stressed (28, 29, 31, 33–37, 40, 44).

#### COVID-19 and SARS

The reluctance to visit the hospital was attributed to a persistent fear of contracting an infection (38). They felt claustrophobic due to the lockdown because they were forced to stay inside their homes (44). This increased abusive relationships, as well as an increase in the frequency and severity of intimate partner violence and family conflicts (42). Fear of joblessness or losing a job, as well as financial hardships as a result of the prolonged lockdown, resulted in depression, anxiety, and gender-based violence (42, 44). Working women felt overwhelmed as they were forced to restructure their routine to strike a balance between work and home chores while remaining at home (44). During SARS, despite their willingness to deliver normally in a public facility, they preferred Cesarean section in a private facility due to the risk of contracting SARS (33).

#### Zika

Most participants expressed concern and fear that their baby would have complications during diagnostic procedures and if born with same would require special care with compromised acceptance (43). Even though healthcare providers performed their duties with dedication, they were also subjected to physical and emotional stress (29, 41). Female health workers explained that their workload was doubled due to a staff shortage, leaving them frustrated (30, 36, 37).

#### Ebola

Women were afraid to visit health facilities because they believed the Ebola virus was being injected by healthcare providers, causing fear of cross-infection (34, 37). Inadequate preparation due to a lack of knowledge resulted in mistrust in midwives, motivating women to avoid health facilities (37).

#### H1N1 Influenza

According to the study, extensive media broadcasting exacerbated hyper-awareness, anxiety, and fear of contracting the infection, as well as unwarranted panic (31). Participants in the higher risk zone expressed more significant concern than those in other containment zones, owing to the increasing number of illnesses and deaths (40).

### Coping Strategies and Support Systems

Women used a variety of coping strategies to protect themselves from the pandemic and its repercussions. They triggered psychological defensive systems, resorted to active or passive psychological tactics, and changed their sleep schedules when under work-related stress (41, 42). Additional measures to ensure routine work include increasing food intake, regular exercise, and preserving physical strength (41).

Female healthcare professionals claimed that family, society, and the goodwill of patients encouraged them to battle the pandemic and brought them happiness (34, 41). Additionally, they reported that social support was critical for feeling valued (41). Pre-employment training aided in the conversion of negative emotions to good ones. Participants expressed considerable support for government directives on social distancing to protect themselves and those in danger. Healthcare providers were a source of information that could be relied upon. Competence in learning, coordinated support, and government-provided prevention and control training equipped them to confront and resolve problems (37). Nurses at work developed coping techniques such as self-mirroring, self-preservation, and self-transcendence. This alleviated fear and infection while providing compassionate treatment in partnership with colleagues (32).

Changes in daily routine and cessation of social activities were cited as coping mechanisms, resulting in a dearth of leisure, social, and outdoor activities (39). Regardless of the epidemic, women used measures such as avoidance, and hygienic practices, social/physical distancing, movement limitation for self and visitors to the home, monitoring the situation via news broadcasts, and modifying/canceling trip plans (30, 31, 33). Some women decided to delay childbearing to prevent putting the child at risk (31, 39, 43). Financial support was stated to have been supplied by the spouse and family to help with the problem (28, 31, 33, 35, 39, 43).

Using COREQ, we presented the quality assessment of the studies (Supplementary Material 1). It is a complete and coherent checklist of 32 items divided into three categories: research team and reflexivity, study design, and findings analysis. Many studies were presented regarding interviewer credentials and the interviewer's relationship with the participants. Almost all of the articles provided comprehensive theoretical frameworks and participant recruitment strategies. All of the studies adhered to the standard guidelines for analysis and reporting. None of the studies used repeat interviews, and only a few studies documented participant non-participation and member-check approaches.

## Discussion

Women's reproductive health is determined to be significantly harmed as a result of impeded reproductive, maternity, and child health care due to human resource constraints, as reported by 66% of nations (45). Disruptions in health services increased demand for home births, either out of fear or insufficient support in institutions (46–48). Lack of knowledge or information, lack of permission from family or husband to access facility, geographical barrier, lack of understanding of prevention methods, and fear of infection were the top barriers women faced in accessing health care (49). Frequent policy changes left end users, and service providers perplexed about how to deal with the situation, resulting in a negative impact on sexual and reproductive health services (50, 51).

Globally, domestic violence against women has escalated, exposing these victims to sexual assault and distress (52). Reduced access to alcohol and tobacco products frequently increased violent behavior, exacerbating domestic abuse. Additionally, a dearth of contraception during pandemics exacerbated undesired pregnancies (53). During emergencies, exposure to hygienic goods has decreased, posing health dangers (54). Job loss has economic consequences, making it even more difficult for women of low social status, particularly in developing countries, to get proper maternal health care; this exacerbates anxiety and grief and increases violence against women (52). Pandemics also exacerbate malnutrition among the poor, owing to food instability, resource constraints, and financial insecurity (55). On the other side, physical activity has been reduced due to the lockdown, resulting in a huge increase in the number of obese wealthy individuals; as a result, the risk of non-communicable disease has increased (56).

Women are disproportionately more likely to experience situations in the workplace that demand them to display resilience when they are underrepresented at the top levels of decision-making (57). A resilient person adjusts and responds to stressors in various ways, including embracing the new reality or improvising through efficient coping mechanisms (58). Individuals may have functional or dysfunctional coping strategies; however, positive coping mechanisms are likely to result in improved resilience; hence, coping and resilience, while distinct, are also related. It is necessary to understand the women's capacity to manage and develop resilience in various situations to address women's vulnerabilities (59). It is critical to examine organizational power systems from a resilience and gender viewpoint. As a result, the female perspective on resilience challenges the mainstream masculine discourse on resilience in the context of pandemics (59).

Despite obstacles, women remained resilient, adapting to their circumstances in various ways. Throughout the pandemic, the avoidance of travel, visiting or meeting new people, and going to work were embraced to cope with the crisis (60, 61). Positive outcomes are associated with problem-solving strategies, whereas emotional coping mechanisms such as blaming, avoiding, and ruminating have negative consequences for depression and anxiety (62). Self-care activities such as hygiene and social support aided in managing the pandemic condition (60, 63).

During pandemics, women are at an increased risk. Furthermore, more than 70% of community health workers worldwide are female. During pandemics, multifactorial challenges are exacerbated in a unique way among women. To combat health disparities during pandemics, gender-informed policies are required. There is a need for a more in-depth understanding of the potentially gendered nature of crisis response and the identification of new research avenues.

Regardless of their roles and responsibilities in society, women faced a slew of health-related challenges during pandemics. Gender inequity during pandemics should not be overlooked in reporting. Women must have equal rights and opportunities, free of discrimination, which necessitates immediate action to improve women's rights and achieve the SDGs. Women's engagement in pandemic-related services should be investigated, as this will aid in the development of strategies to reduce vulnerabilities.

## Author Contributions

MRS and SD designed the systematic review under the supervision of KCS. MRS and SD synthesized and analyzed the data, drafted manuscript, and revised the drafts based on comments from other co-authors. KCS and SP reviewed the various versions of this manuscript and provided constructive comments. All authors read and approved the final manuscript.

## Conflict of Interest

The authors declare that the research was conducted in the absence of any commercial or financial relationships that could be construed as a potential conflict of interest.

## Publisher's Note

All claims expressed in this article are solely those of the authors and do not necessarily represent those of their affiliated organizations, or those of the publisher, the editors and the reviewers. Any product that may be evaluated in this article, or claim that may be made by its manufacturer, is not guaranteed or endorsed by the publisher.
