# Supplementary material for: Health-Related Challenges and Coping Strategies Among Women During Pandemics: A Systematic Review of Qualitative Studies
Source: Front Health Serv. 2022 Apr 15;2:847753. doi: 10.3389/frhs.2022.847753 (PMC10012754; doi:10.3389/frhs.2022.847753)
Supplement: Supplementary file 1 [file Data_Sheet_1.pdf]

Supplementary Material

Supplementary file 1: Quality assessment of the studies using consolidated criteria for reporting qualitative research (COREQ) assessment tool

	Anderson et al. 2020	Arias et al.2020	Belizan et al. 2020	Boyd 2012	Chiang et al.2007	Dodgson et al.2010	Erland and Dahl 2017	Flowers et al. 2014	Gomez et al.2020	Jones et al.2017	Homer et al. 2020	Linde &Siqueira 2018	Lynch et al.2012	Niuniu et al. 2020	Sabri et al. 2020	Tirado et al.2020	Williams et al. 2020
**Research team and reflexivity**																	
Personal characteristics																	
Interviewer	●	●	●	●	×	●	●	×	●	●	×	×	●	×	●	●	●
Credentials	×	×	●	×	×	×	●	×	●	●	×	×	×	●	●	●	●
Occupation	×	×	×	×	×	●	×	×	×	●	×	×	×	×	●	×	●
Gender	×	●	●	×	×	●	●	×	●	×	×	×	×	×	×	×	●
Experience and training	×	×	●	×	×	●	×	×	×	●	×	×	×	●	×	●	×
Relationship with participants																	
Relationship established	●	×	×	×	×	×	●	×	×	●	×	×	×	×	×	×	×
Participant knowledge of the interviewer	×	×	×	×	×	×	×	×	●	×	×	×	×	●	×	×	×
Interviewer characteristics	×	●	×	×	×	×	×	×	●	×	●	×	×	×	×	×	×
Theoretical Framework																	
Methodological orientation	●	●	●	●	●	●	●	●	●	●	×	●	●	●	●	●	●
Participant selection			●								●						
Sampling	●	●	●	●	●	●	●	●	●	●	●	●	●	×	●	●	●
Method of approach	●	●	●	●	×	●	●	●	●	●	●	×	×	×	●	●	●
Sample size	●	●	●	●	●	●	●	●	●	●	●	●	●	●	●	●	●
Non-participation	●	×	×	×	×	×	×	×	●	×	×	×	×	×	×	●	×
Setting																	
Setting of data collection	●	●	●	●	●	●	●	●	●	●	●	×	×	●	●	●	●
Presence of non-participants	×	×	●	●	×	×	●	×	●	×	×	×	×	×	×	×	×
Description of sample	●	●	●	●	●	●	●	●	●	●	●	●	●	●	●	●	●
Data collection																	
Interview guide	●	●	●	●	×	●	×	●	●	●	●	●	●	●	●	●	●
Repeat interviews	×	×	●	×	×	×	×	×	×	×	×	×	×	●	×	×	×
Audio/visual recording	●	●	●	●	●	●	●	●	●	●	×	●	●	●	●	●	●
Field notes	●	×	●	●	×	●	●	×	●	×	×	×	●	●	●	●	●
Duration	×	●	×	●	●	●	●	×	●	●	●	●	●	●	×	●	●
Data saturation	×	×	●	×	×	●	×	×	×	×	●	×	×	●	●	●	×
Transcripts returned	×	×	×	×	×	×	×	×	×	×	×	×	×	×	×	×	×
**Analysis and findings**																	
Number of data coders	×	●	×	●	×	●	●	●	×	●	×	×	●	●	●	●	●
Descriptions of the coding tree	×	●	●	×	×	×	×	×	●	●	×	×	×	×	●	×	×
Derivation of themes	●	●	×	●	×	×	●	●	●	●	●	×	●	●	●	●	●
Software	×	●	●	●	×	●	×	●	●	●	×	●	●	×	●	●	●
Participant checking	×	×	×	×	×	×	×	×	×	×	×	×	×	×	×	●	×
Reporting																	
Quotations presented	●	●	●	●	●	●	●	●	●	●	●	●	×	●	●	●	●
Data and findings consistent	●	●	●	●	●	●	●	●	●	●	●	●	×	●	●	●	●
Clarity of major themes	●	●	●	●	●	●	●	●	●	●	●	●	×	●	●	●	●
Clarity of minor themes	●	●	●	●	●	●	●	●	●	●	●	●	●	●	●	●	●

● Represents addressed the point, and × represents not addressed the points
